# Traumatic Anterior Dislocation of the Shoulder: Factors Affecting the Progress of the Traumatic Anterior Dislocation

**DOI:** 10.4055/cios.2009.1.4.188

**Published:** 2009-11-25

**Authors:** Yong Girl Rhee, Nam Su Cho, Seung Hyun Cho

**Affiliations:** Department of Orthopaedic Surgery, Kyung Hee University School of Medicine, Seoul, Korea.

**Keywords:** Shoulder dislocation, Traumatic, Anterior, Prognostic factor

## Abstract

**Background:**

The aim of this study was to identify the factors that affect the progress of a traumatic anterior dislocation of the shoulder.

**Methods:**

Two hundred and thirty-eight patients (246 shoulders) with a traumatic anterior dislocation were enrolled in this study. The mean age at the time of surgery was 25 years (range, 14 to 47 years). There were 214 men and 24 women.

**Results:**

One hundred and sixty-four shoulders (67%) were younger than 20 years at the time of the first dislocation. Patients younger than 20 years showed a shorter interval of redislocation (*p* = 0.001) and a higher frequency of dislocation (*p* = 0.001). Athletic patients experienced their first dislocation at a younger age (*p* = 0.023) and showed a shorter interval of redislocation (*p* = 0.001) than their non-athetic counterparts. The incidence of classic and non-classic Bankart lesions was unaffected by age at the time of the first dislocation, interval between the first and second dislocation or the frequency of dislocation. Patients with bony Bankart lesions had a higher frequency of dislocation (*p* = 0.043).

**Conclusions:**

The age at the time of the first dislocation and athletic activity were related to early redislocation and a high frequency of dislocation. Bony Bankart lesions were observed more often in patients with a higher frequency of dislocation. Early surgical treatment is a good option for young athletic patients with a bony Bankart lesion and a short interval between the first and second dislocation.

A shoulder joint dislocation is the most common dislocation in human joints. However, the treatment of a first-time traumatic shoulder dislocation is still controversial due to the high frequency of recurrence.

Many studies on the factors that affect redislocation report that the most important factor is the patient's age at the time of the first dislocation.^1^-4) Other factors, such as the patient's athletic activity,^5^-7) associated pathologic conditions^8^-12) and method of immobilization,^13^-14) can also affect redislocation.

This study examined the preoperative prognostic factors to determine the natural history of traumatic shoulder dislocation and clarify the treatment guidelines. We hypothesized that 1) the interval between dislocations is shorter in younger patients at the time of the first dislocation, and 2) the size and condition of the lesions becomes more severe as the frequency of redislocation increases.

## METHODS

Two hundred and eighty-seven shoulders with a Bankart repair of the anterior shoulder instability were identified from January 2000 through to December 2002. The preoperative medical records and other relevant information from each patient were retrieved and reviewed. Those who had associated fractures of the proximal humerus, rotator cuff tear, previous shoulder operations, and glenohumeral or acromioclavicular arthritis were excluded. In total, two hundred and forty-six shoulders in two hundred and thirty-eight patients were examined. There were 214 men and 24 women. The average age at the time of surgery was 25 years (range, 14 to 47 years). Arthroscopic repair and open repair was performed in 130 and 116 shoulders, respectively.

A retrospective review of the prospectively collected data was conducted. The preoperative patient chart, radiographs, surgical records, and intraoperative arthroscopic photographs of all patients were analyzed. Age at the time of the first dislocation, the frequency of dislocation, interval between the first and second dislocation, activity of the patient and intraarticular pathologies observed during surgery were identified as prognostic factors that can affect the progress of traumatic dislocation. The relationships between factors were also analyzed. All procedures were performed by the same surgeon. The age at the time of the first dislocation, frequency of dislocation and interval between the first and second dislocation were analyzed by reviewing the surgical; medical records and by a telephone interview. Along with a reassessment of the medical records and an analysis of the arthroscopic photographs, the intraarticular pathologies were classified as classic Bankart lesions or non-classic Bankart lesions. Non-classic Bankart lesions included: Bony Bankart lesions, humeral avulsion of the glenohumeral ligament (HAGL),15) an anterior labroligamentous periosteal sleeve avulsion (ALPSA)-like lesions,16) and midsubstance tears. The manifestations of each lesion were examined to determine if it affected the natural history of shoulder dislocation. The presence of bony Bankart lesions, shown in preoperative radiological studies, and the size of the Hill-Sachs lesions were also considered to be contributing factors.

### Statistical Analysis

Spearman's correlation and chi-square tests were used to analyze the age at the time of the first dislocation, frequency of dislocation and the interval between dislocations. A Kruskall-Wallis test was used to determine if each prognostic factor was affected by age, frequency of dislocation and interval between dislocations. The presence of bony Bankart lesions and the athletic activity were analyzed by a Mann-Whitney U test. The SPSS ver. 12.0 (SPSS Inc., Chicago, IL, USA) was used for all statistical analyses, with an α level set to 0.05.

## RESULTS

### Age at the Time of the First Dislocation

Table 1 lists the data associated with age at the time of the first dislocation. The average age at the time of the first dislocation was 19.9 ± 5.9 years (range, 4 to 41 years). The frequency of redislocation decreased with increasing age of the patients at the time of the first dislocation (*p* < 0.001). The patients who had experienced the first dislocation when they were younger than 20 years had a higher frequency of redislocation than those over 20 years (*p* < 0.001). When comparing each age group according to the interval between the first and second dislocation, the 16-20 years group had the shortest interval. The interval between dislocations increased with increasing age of the patients at the time of their first dislocation (*p* < 0.001). No significant relationships were observed between the patient's age at the time of the first dislocation and the frequency of classic or non-classic Bankart lesions (*p* = 0.434). In addition, the existence of bony Bankart lesions (*p* = 0.248) and the size of Hill-Sachs lesions (*p* = 0.625) were not affected by the patient's age at the time of the first dislocation.

### Frequency of Dislocation

Table 2 lists the frequency of dislocation, related to age at the time of the first dislocation and the interval between the first and second dislocation. The average frequency of dislocation was 23.7 ± 28.8 times (range, 1 to 150 times). One hundred and one shoulders experienced more than 16 pre-operative dislocations (41.1%). The group with the highest frequency of redislocation was the group that experienced the first dislocation at the youngest age (*p* < 0.001). Significantly, in the group that experienced a dislocation less than 5 times, the interval between the first and second dislocation was comparatively longer than those of the other groups (*p* = 0.043).

Another significant difference was in the group that experienced more than 16 dislocations. Eighteen shoulders in this group had preoperative bony Bankart lesions (64% of total bony Bankart lesion) (*p* = 0.034). However, the size of the Hill-Sachs lesions (*p* = 0.192) and the existence of classic or non-classic Bankart lesions (*p* = 0.337) did not affect the frequency.

### Interval between the First and Second Dislocation

Table 3 shows the data associated with the interval between the first and second dislocation. The average interval between the first and second dislocation was 29.1 ± 39.3 weeks (range, 0 to 192 weeks). Of the two hundred and forty-six shoulders, 54 shoulders (22.0%) experienced a redislocation within 3 weeks from the first dislocation; 78 shoulders (31.7%) from 3 weeks to 3 months; and 83 shoulders (33.7%) from 3 months to 1 year. In the case that the interval between the first and second dislocations was longer than 1 year, the age at the time of the first dislocation was comparably older (*p* = 0.043). The frequency of redislocation decreased as the interval between dislocations was longer (*p* = 0.006). Non-classic Bankart lesions were less frequent in the group with an interval between dislocations of longer than 1 year than in the other groups (*p* = 0.043). The size of the Hill-Sachs lesions (*p* = 0.192) and the existence of classic or non-classic Bankart lesions (*p* = 0.337) did not affect the interval between dislocations.

### Athletes

Fifty-four patients (22.7%) were athletes, with 25 collisions and 29 noncollision. Seventeen patients (31.5%) played sport at a competitive, recreational level. In athletes, the mean age at the time of the first dislocation was 18.4 years. The mean total number of dislocations was 23.4 and the mean interval from the first to the second dislocation was 13.1 weeks (Table 4). Compared to non-athletes, the athletes were generally younger at the time of the first dislocation (*p* = 0.023) and the interval between the first and second dislocation was shorter (*p* = 0.001), but there was no difference in the total number of dislocations between athletes and non-athletes (*p* = 0.940). Non-classic Bankart lesions were observed in 31.5% (17 shoulders) of athletes and 26.6% (51 shoulders) of non-athletes. Bony Bankart lesions were present in 9.3% (5 shoulders) of athletes and 12.0% (23 shoulders) of non-athletes. Therefore, athletic activity may not affect the manifestations of the lesions. The size of the Hill-Sachs lesions in the athletes and non-athletes groups was similar.

### Intraoperative Lesions

One hundred and seventy-eight (72.3%) out of 246 shoulders had classic Bankart lesions and 68 shoulders (27.7%) had non-classic Bankart lesions. Among the non-classic Bankart lesions, 6.1% (15 shoulders), 2.5% (6 shoulders), 7.7% (19 shoulders) and 11.4% (28 shoulders) were ALPSA lesions, HAGL lesions, midsubstance tears and bony Bankart lesions, respectively. The identification of classic or non-classic Bankart lesions was not related to the age at the time of the first dislocation (*p* = 0.484), frequency (*p* = 0.221) or interval (*p* = 0.888) (Table 5). The patient's age at the time of the first dislocation and the interval between the first and second dislocation were also not affected by the existence of bony Bankart lesions. However, the total frequency of dislocations increased when bony Bankart lesions were present (*p* = 0.043) (Table 6).

## DISCUSSION

A dislocation of the shoulder is the most common of all joint dislocations and a recurrent dislocation is the main complication after an acute traumatic anterior dislocation of the shoulder. Hovelius^17^,18) reported a 1.7% incidence of traumatic dislocation among the general population in Sweden and 8% among Swedish hockey players in the highest league. According to Simonet and Cofield6) and Norlin,19) the incidence in a group of 20 to 30 years individuals ranged from 13 to 18 per 100,000 inhabitants. Based on these and more recent studies, the redislocation rate ranges from 4% to 96% depending on a variety of factors, such as age, athletic activity and associated lesions.

Among those factors, the patient's age at the time of the first dislocation was found to be the most important contributing factor in redislocation. The rate of redislocation increased with decreasing age of the patients at the time of the first dislocation. McLaughlin and MacLellan3) found that 95% of 181 primary dislocation patients aged 11 to 20 years experienced a redislocation. Rowe et al.4) also found, in a series of 488 patients who had both a primary and recurrent dislocation, an 83% recurrence rate in 107 shoulders of patients who were younger than 20 years. Meanwhile, Hovelius et al.2) reported a 47% rate of redislocation at the 2-year follow up of 102 primary traumatic dislocation in the under 22 years group. At the 5-year follow up, the rate increased to 64% and 67% at the 10-year follow up. In a group of 60 shoulders in patients aged from 23 to 29 years, the redislocation rate was 28%, 48% and 58% at the 2-, 5- and 10-year follow up, respectively. In a group of 95 shoulders in patients aged 30 to 40 years, the rate of redislocation was 13%, 20%, and 21% at the 2-, 5-, and 10-year follow up, respectively. Although previous reports indicated that the rate of redislocation increased with decreasing patient's age at the time of the first dislocation, unsolved questions still remained regarding the relationships between age and the frequency of dislocation, as well as between the frequency and interval of redislocation.

In this study, the results showed that as a patient was younger at the time of the first dislocation, the frequency of a redislocation increased. Indeed, the group under 20 years experienced a 3 times higher frequency of redislocation than the over 20 years group. The intervals differed from 59 weeks in the over 26 years group to 18 weeks in the 16 to 20 years group. This large difference provides strong evidence that a dislocation in people under 20 years will redislocate with a short interval. On the other hand, patients who had longer intervals between the first and the second dislocation showed a comparably less frequent rate of redislocation.

Athletic activity is another indicator that can influence a redislocation. Simonet and Cofield6) found an 80% of redislocation rate in athletes under 20 years and 30% in non-athletes in the same age group. Wheeler et al.7) recommended arthroscopic treatment for younger athletes after the initial dislocation due to the 92% rate of redislocation in young athletes who were treated conservatively. Arciero et al.5) reported details of 36 shoulders of athletes (mean age 20 years) who had experienced traumatic first-time anterior dislocation. Eighty percent (15 shoulders) of the conservatively treated group experienced a redislocation while only 14% (21 shoulders) of the arthroscopic treatment group experienced a redislocation. Therefore, they reported that surgical treatment should be suggested to patients under 20 years who experience a dislocation in order to prevent recurrence. te Slaa et al.20) noted that recurrence took place in 64% of patients younger than 20 years of age and in 6% of those older than 40 years, but recurrence was unrelated to the activity of the patient.

In this study, athletes experienced their first dislocation at a comparatively younger age than non-athletes, and had a significantly shorter interval between the first and second dislocation. However, once a redislocation occurred, the rate of recurrence in athletes and non-athletes was similar.

The type of lesions, classic or non-classic Bankart lesions, had no relationship with the patient's age at the time of the first dislocation. It might be believed that athletes would have more severe lesions in the capsulolabral complex as the redislocation rates increased. However, the results showed that the presence of classic and non-classic Bankart lesions were unrelated to the redislocation rates. Another unexpected outcome is in the group with non-classic Bankart lesion and bony Bankart lesion. Due to the excess exercise and repetition of certain postures, it was believed that athletes would experience more frequent redislocations, resulting in non-classic Bankart lesions, particularly bony Bankart lesions. However, there was no difference in these categories between athletes and non-athletes.

In summary, the age, activity, frequency and interval of dislocation have close relationships with the redislocation rates, but the presence of non-classic Bankart lesions or bony Bankart lesions are unrelated to the redislocation rates. It is possible that the type of Bankart lesion and the severity of bony Bankart lesions are determined at the first dislocation. Remarkably, patients with bony Bankart lesions demonstrated a high frequency of dislocation. Therefore, patients who present with bony Bankart lesions in radiography are likely to have recurrent shoulder instability after the dislocation.

In order to predict the natural history of traumatic anterior dislocation of the shoulder, more objective and various cases will need to be analyzed. However, this study is limited by its retrospective nature. For further study into the natural history of shoulder dislocation, it will be important to examine the progress of shoulder dislocations related to the level of force causing the injury, how immobilization relates to the total number of dislocations and the interval between the dislocations as well as how adequate rehabilitation affects the progress of dislocation.

In this study, the age at the time of first dislocation and athletic activity were related to an early redislocation and a high frequency of dislocation. Bony Bankart lesions were found more often in patients showing a higher frequency of dislocation. Early surgical treatment would be a good option for athletic patients who are under 20 years if they present with bony Bankart lesions after the first dislocation and have a short interval of recurrence despite the conservative treatment.

It is possible that various lesions of anterior shoulder instability are determined at the time of the first dislocation because the type of lesions were less affected by prognostic factors, such as age, frequency of dislocation and interval between dislocations.

## Figures and Tables

**Table 1:**
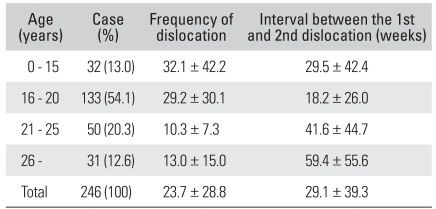
Data Associated with the Age at the Time of First Dislocation

**Table 2:**
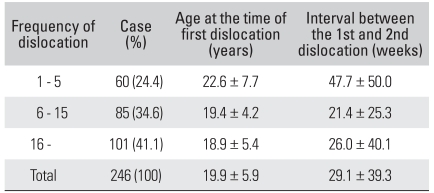
Data Associated with the Frequency of Dislocation

**Table 3:**
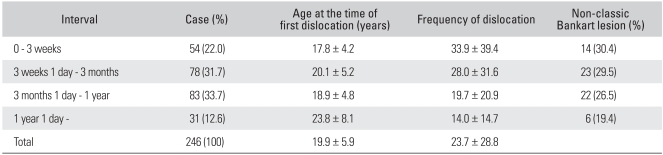
Data Associated with the Interval between the First and Second Dislocation

**Table 4:**
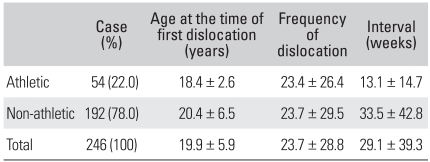
Data Associated with the Patient's Activity

**Table 5:**
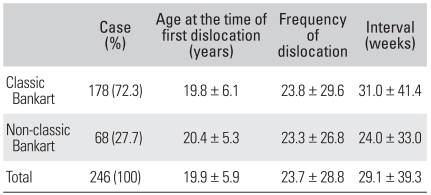
Data Associated with the Type of Bankart Lesion

**Table 6:**
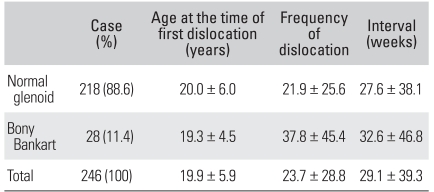
Age, Frequency, and Interval of Dislocation according to the Glenoid Pathology
